# Guidelines and definitions for research on epithelial–mesenchymal transition

**DOI:** 10.1038/s41580-020-0237-9

**Published:** 2020-04-16

**Authors:** Jing Yang, Parker Antin, Geert Berx, Cédric Blanpain, Thomas Brabletz, Marianne Bronner, Kyra Campbell, Amparo Cano, Jordi Casanova, Gerhard Christofori, Shoukat Dedhar, Rik Derynck, Heide L. Ford, Jonas Fuxe, Antonio García de Herreros, Gregory J. Goodall, Anna-Katerina Hadjantonakis, Ruby Y. J. Huang, Chaya Kalcheim, Raghu Kalluri, Yibin Kang, Yeesim Khew-Goodall, Herbert Levine, Jinsong Liu, Gregory D. Longmore, Sendurai A. Mani, Joan Massagué, Roberto Mayor, David McClay, Keith E. Mostov, Donald F. Newgreen, M. Angela Nieto, Alain Puisieux, Raymond Runyan, Pierre Savagner, Ben Stanger, Marc P. Stemmler, Yoshiko Takahashi, Masatoshi Takeichi, Eric Theveneau, Jean Paul Thiery, Erik W. Thompson, Robert A. Weinberg, Elizabeth D. Williams, Jianhua Xing, Binhua P. Zhou, Guojun Sheng, On behalf of the EMT International Association (TEMTIA)

**Affiliations:** 1 Departments of Pharmacology and Pediatrics, Moores Cancer Center, University of California, San Diego, La Jolla, CA USA; 2 Department of Cellular and Molecular Medicine, University of Arizona, Tucson, AZ USA; 3 Molecular and Cellular Oncology Lab, Department of Biomedical Molecular Biology, Ghent University, Cancer Research Institute Ghent (CRIG), VIB Center for Inflammation Research, Ghent, Belgium; 4 Laboratory of Stem Cells and Cancer, Université Libre de Bruxelles, Bruxelles, Belgium; 5 Department of Experimental Medicine 1, Nikolaus-Fiebiger-Center for Molecular Medicine, Friedrich-Alexander-University Erlangen-Nürnberg, Erlangen, Germany; 6 Division of Biology and Biological Engineering, California Institute of Technology, Pasadena, CA USA; 7 Department of Biomedical Science and Bateson Centre, University of Sheffield, Sheffield, UK; 8 Departamento de Bioquímica, Universidad Autónoma de Madrid (UAM), Instituto de Investigaciones Biomédicas ‘Alberto Sols’ (CSIC-UAM), IdiPAZ & Centro de Investigación Biomédica en Red de Cáncer (CIBERONC), Madrid, Spain; 9 Institute for Research in Biomedicine (IRB Barcelona), Barcelona Institute of Science and Technology/Institut de Biologia Molecular de Barcelona (IBMB-CSIC), Barcelona, Spain; 10 Department of Biomedicine, University of Basel, Basel, Switzerland; 11 Department of Biochemistry and Molecular Biology, University of British Columbia and British Columbia Cancer Research Centre, Vancouver, BC Canada; 12 Departments of Cell and Tissue Biology, and Anatomy, University of California at San Francisco, San Francisco, CA USA; 13 Department of Pharmacology, University of Colorado Anschutz Medical Campus, Aurora, CO USA; 14 Department of Laboratory Medicine (LABMED), Division of Pathology, Karolinska University Hospital and Department of Microbiology, Tumor and Cell Biology (MTC), Karolinska Institutet, Stockholm, Sweden; 15 Programa de Recerca en Càncer, Institut Hospital del Mar d’Investigacions Mèdiques (IMIM) and Departament de Ciències Experimentals i de la Salut, Universitat Pompeu Fabra, Barcelona, Spain; 16 Centre for Cancer Biology, An alliance of SA Pathology and University of South Australia, Adelaide, SA Australia; 17 Developmental Biology Program, Sloan Kettering Institute, Memorial Sloan Kettering Cancer Center, New York, NY USA; 18 School of Medicine, College of Medicine, National Taiwan University, Taipei, Taiwan; 19 Department of Medical Neurobiology, Institute for medical Research Israel-Canada and the Safra Center for Neurosciences, Hebrew University of Jerusalem, Hadassah Medical School, Jerusalem, Israel; 20 Department of Cancer Biology, Metastasis Research Center, MD Anderson Cancer Center, Houston, TX USA; 21 Department of Molecular Biology, Princeton University, Princeton, NJ USA; 22 Centre for Cancer Biology, an Alliance of SA Pathology and the University of South Australia, Adelaide, SA Australia; 23 Department of Physics, Northeastern University, Boston, MA USA; 24 Department of Anatomic Pathology, The Division of Pathology and Laboratory Medicine, University of Texas MD Anderson Cancer Center, Houston, TX USA; 25 Department of Medicine (Oncology) and Department of Cell Biology and Physiology, ICCE Institute, Washington University, St. Louis, MO USA; 26 Department of Translational Molecular Pathology, University of Texas MD Anderson Cancer Center, Houston, TX USA; 27 Cancer Biology and Genetics Program, Sloan Kettering Institute, Memorial Sloan Kettering Cancer Center, New York, NY USA; 28 Department of Cell and Developmental Biology, University College London, London, UK; 29 Department of Biology, Duke University, Durham, NC USA; 30 Departments of Anatomy and Biochemistry/Biophysics, University of California, San Francisco, School of Medicine, San Francisco, CA USA; 31 Murdoch Children’s Research Institute, Royal Children’s Hospital, Parkville, VIC Australia; 32 Instituto de Neurociencias (CSIC-UMH) Avda Ramon y Cajal s/n, Sant Joan d´Alacant, Spain; 33 Université Claude Bernard Lyon 1, INSERM 1052, CNRS 5286, Centre Léon Bérard, Cancer Research Center of Lyon, Lyon, France; 34 Department of Cellular and Molecular Medicine, University of Arizona, Tucson, AZ USA; 35 INSERM UMR 1186, Integrative Tumor Immunology and Genetic Oncology, Gustave Roussy, University Paris-Saclay, Villejuif, France; 36 Department of Medicine, Perelman School of Medicine, University of Pennsylvania, Philadelphia, PA USA; 37 Department of Zoology, Graduate School of Science, Kyoto University, Kyoto, Japan; 38 RIKEN Center for Biosystems Dynamics Research, Kobe, Japan; 39 Centre de Biologie du Développement (CBD), Centre de Biologie Intégrative (CBI), Université de Toulouse, CNRS, UPS, Toulouse, France; 40 Guangzhou Regenerative Medicine and Health, Guangdong Laboratory, Guangzhou, China; 41 School of Biomedical Sciences and Institute of Health and Biomedical Innovation, Queensland University of Technology, Translational Research Institute, Woolloongabba, QLD Australia; 42 Whitehead Institute for Biomedical Research, Department of Biology, MIT Ludwig Center for Molecular Oncology, Massachusetts Institute of Technology, Cambridge, MA USA; 43 Australian Prostate Cancer Research Centre-Queensland (APCRC-Q) and Queensland Bladder Cancer Initiative (QBCI), School of Biomedical Sciences and Institute of Health and Biomedical Innovation, Queensland University of Technology, Woolloongabba, QLD Australia; 44 Department of Computational and Systems Biology and UPMC-Hillman Cancer Center, University of Pittsburgh, Pittsburgh, PA USA; 45 Department of Molecular and Cellular Biochemistry and UK Markey Cancer Center, University of Kentucky College of Medicine, Lexington, KY USA; 46 International Research Center for Medical Sciences (IRCMS), Kumamoto University, Kumamoto, Japan; 47 Present Address: Institut Curie, PSL Research University, Paris, France

**Keywords:** Cancer, Developmental biology, Epithelial-mesenchymal transition

## Abstract

Epithelial–mesenchymal transition (EMT) encompasses dynamic changes in cellular organization from epithelial to mesenchymal phenotypes, which leads to functional changes in cell migration and invasion. EMT occurs in a diverse range of physiological and pathological conditions and is driven by a conserved set of inducing signals, transcriptional regulators and downstream effectors. With over 5,700 publications indexed by Web of Science in 2019 alone, research on EMT is expanding rapidly. This growing interest warrants the need for a consensus among researchers when referring to and undertaking research on EMT. This Consensus Statement, mediated by ‘the EMT International Association’ (TEMTIA), is the outcome of a 2-year-long discussion among EMT researchers and aims to both clarify the nomenclature and provide definitions and guidelines for EMT research in future publications. We trust that these guidelines will help to reduce misunderstanding and misinterpretation of research data generated in various experimental models and to promote cross-disciplinary collaboration to identify and address key open questions in this research field. While recognizing the importance of maintaining diversity in experimental approaches and conceptual frameworks, we emphasize that lasting contributions of EMT research to increasing our understanding of developmental processes and combatting cancer and other diseases depend on the adoption of a unified terminology to describe EMT.

## Abstract (web summary)

In this Consensus Statement, the authors (on behalf of the EMT International Association) propose guidelines to define epithelial–mesenchymal transition, its phenotypic plasticity and the associated multiple intermediate epithelial–mesenchymal cell states. Clarification of nomenclature and definitions will help reduce misinterpretation of research data generated in different experimental model systems and promote cross-disciplinary collaboration.

## Introduction

**Fig. 1:**
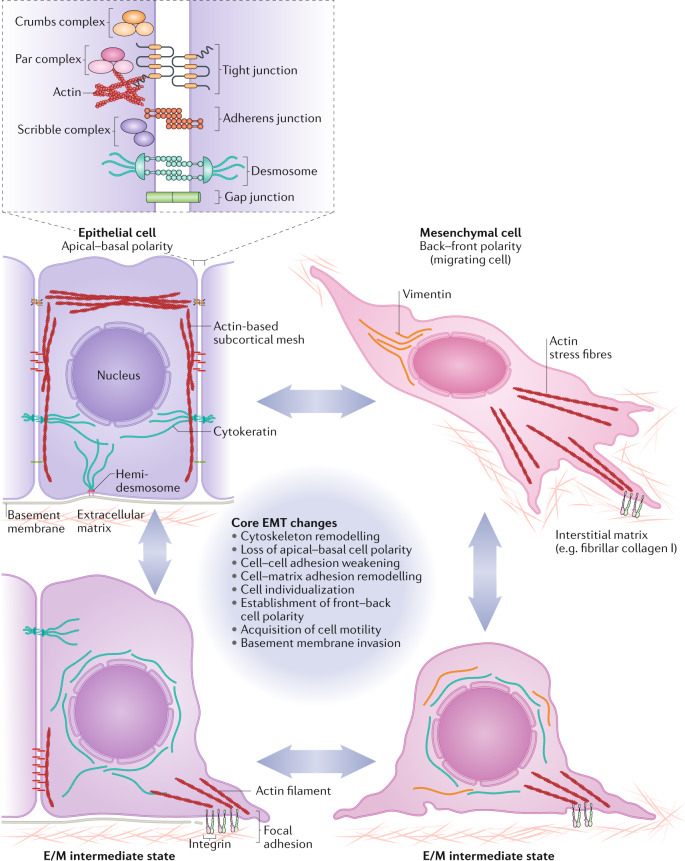
EMT diversity represented by an epithelial–mesenchymal plasticity model. Various cellular features associated with an epithelial or a mesenchymal cell state are found in a range of combinations and to different degrees in cells in different developmental contexts. Epithelial cells are connected with each other via a variety of epithelial cell junctions, including adherens junctions, desmosomes, gap junctions and tight junctions. Adherens junctions are connected to cortical actin bundles, while desmosomes are linked with cytokeratin intermediate filaments. Tight junctions are localized at the apical-lateral contact points in order to help maintain epithelial polarity. Apical–basal polarity guides proper organization of the tight junctions, adherens junctions and desmosomes in epithelial cells. Polarity complexes, including the Par, Crumbs and Scribble complexes, define the apical versus basolateral domains of an epithelial cell. Epithelial cells are attached to the underlying basement membrane via hemidesmosomes, which contain integrin to allow binding to the basement membrane and are also linked to cytokeratins inside the cell. By contrast, mesenchymal cells do not contain functional epithelial junctions and present a back–front polarity in their actin stress fibres. Mesenchymal cells contain vimentin-based intermediate filaments and utilize integrin-containing focal adhesions to attach to the extracellular matrix. The accumulated loss or gain of epithelial/mesenchymal (E/M) characteristics pushes a cell towards various intermediate states (bottom left and right) in a fluid and reversible manner, between a complete epithelial (middle left) and a complete mesenchymal (middle right) state. EMT, epithelial–mesenchymal transition.

Epithelial–mesenchymal transition (EMT) is a cellular process during which epithelial cells acquire mesenchymal phenotypes and behaviour following the downregulation of epithelial features. EMT is triggered in response to signals that cells receive from their microenvironment. The epithelial state of the cells in which EMT is initiated is characterized by stable epithelial cell–cell junctions, apical–basal polarity and interactions with basement membrane. During EMT, changes in gene expression and post-translational regulation mechanisms lead to the repression of these epithelial characteristics and the acquisition of mesenchymal characteristics. Cells then display fibroblast-like morphology and cytoarchitecture, as well as increased migratory capacity. Furthermore, these now migratory cells often acquire invasive properties (Fig. 1).

EMT was first described by researchers studying early embryogenesis as a programme with well-defined cellular features^1,2^. It is now widely accepted that EMT occurs normally during early embryonic development, to enable a variety of morphogenetic events, as well as later in development and during wound healing in adults. Moreover, EMT is known to be activated during cancer pathogenesis and tissue fibrosis. The reverse process, known as mesenchymal–epithelial transition (MET), also occurs frequently during development. A salient characteristic of EMT occurring in vivo, whether during normal development or in a pathological context, is that the transition from an epithelial to a mesenchymal state is often incomplete, resulting in cells that reside in intermediate states that retain both epithelial and mesenchymal characteristics. Importantly, these intermediate states can be diverse, depending on the biological context^3^.

**Fig. 2:**
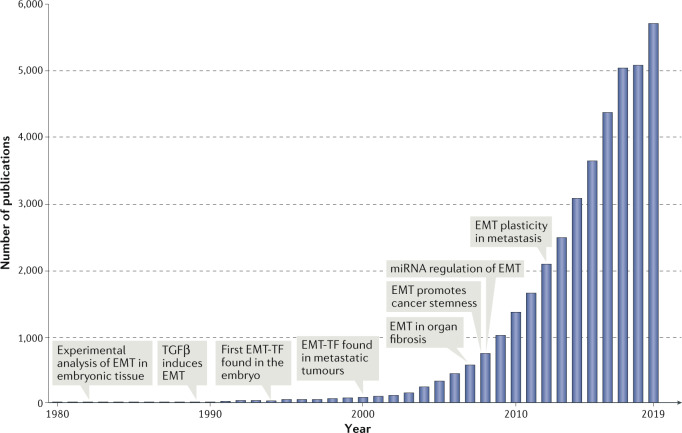
Growth of the primary literature in EMT. The first experimental analysis of epithelial–mesenchymal transition (EMT) in development was published in 1979. The relationship of EMT to growth factors was found in 1989. Transcriptional regulation of EMT was identified in 1994. Subsequent growth of such research was stimulated by linkage of EMT to metastasis, organ fibrosis and stem cells. Growth in the field has been logarithmic since the first TEMTIA meeting in 2003. The graph indicates primary papers published each year, identified by a search of the Web of Science database. The total numbers of publications in 2018 and 2019 exceeded 5,000 and 5,700 articles, respectively. EMT-TF, EMT-associated transcription factors.

The EMT research field has grown explosively over the past 20 years. More than half of all articles on EMT have been published in the past 5 years alone (Fig. 2), and half of those have reported on studies of EMT in the context of cancer biology. The growing complexity and diversity of the EMT literature has resulted in vague and often confusing definitions of EMT and associated nomenclature. Cell biologists have traditionally focused on the microscopically visible and profound changes in cell–cell interactions, cell motility, cytoskeletal organization, cell proliferation and resistance to various stressors that occur during EMT. Molecular biologists have focused on changes in the activity of EMT-associated transcription factors (EMT-TFs) and in aspects of their regulation, often involving various chromatin modifications to orchestrate changes in EMT-associated gene expression. Cancer biologists have often emphasized the acquisition of various malignancy-associated cell phenotypes, notably invasiveness, as well as dissemination and different degrees of cell responsiveness to various therapeutic modalities. However, these context-specific, or even research-community-specific, criteria to define or refer to EMT do not reflect the current experimental shortcomings in the ability to identify EMT events. As we learn more about EMT-associated changes, it is becoming apparent that there is a great diversity of EMT phenotypic manifestations. Thus, narrow definitions have become inappropriate or inaccurate, and a more encompassing definition is required in order to describe this complex cell biological programme.

## Purpose of this Consensus Statement

The use of the term EMT in research areas as diverse as developmental biology, cell biology, tissue homeostasis and disease (notably cancer and fibrosis) has created discrepancies in data interpretation and persistent disagreements about whether the process studied is EMT^4–9^, largely because the plasticity and heterogeneity of EMT programmes have been insufficiently considered. EMT was originally described as an important process in embryonic development during which epithelial cells underwent a phenotypic transformation to mesenchymal cells. These early analyses and subsequent validations in cell culture described the phenotypic changes associated with EMT using a limited set of molecular markers. However, the later identification of EMT as a crucial programme in cancer progression^10,11^ indicated that EMT involves more than the originally identified developmental EMT programmes and that, clearly, many different variations of the EMT programme exist that cannot be accurately defined by those limited sets of markers.

As the complexity of EMT events and EMT regulators in both development and cancer becomes increasingly appreciated, there is a need for the community of researcher experts on EMT to agree on a number of key points. These include: definitions of major EMT-related terms, a description of EMT-associated phenomena, a description of the diverse versions of EMT that can occur in different contexts, the context-dependent function and activity of EMT regulators, and the relationship between the core and non-core EMT functions of EMT-TFs. In the context of cancer, there is also a need to further consider the contributions of genetic alterations, the complex input of changing tumour environments and the EMT-like changes that occur in non-epithelial cancers such as melanoma, sarcoma and leukaemia. Provoked by the passionate town hall discussions that took place during the 2017 and 2019 meetings of the EMT International Association (TEMTIA), TEMTIA proposes the following guidelines to define the EMT programme, its phenotypic plasticity and the resulting multiple intermediate epithelial–mesenchymal states. By building such a consensus on EMT-related concepts, we aim to eliminate semantic problems in the EMT debate and facilitate genuine cross-disciplinary discussion of the roles of EMT in both normal development and pathological conditions.

## A brief history of EMT

Modern EMT studies began with research aimed at understanding tissue morphogenesis during development, cell behaviour in culture and carcinoma invasiveness in cancer progression. Elizabeth Hay recognized the importance of EMT in embryogenesis^1^ and began to discuss the concept of “epithelial–mesenchymal transformation” in the late 1970s. EMT was subsequently observed in the context of neural crest formation^12,13^, heart valve formation^14^ and Müllerian duct regression^15^, as well as in epithelial tissue explants in vitro^16^. This ‘epithelial–mesenchymal transformation’ process was alternately referred to as ‘epithelial–mesenchymal transition’, to distinguish it from the process of neoplastic transformation commonly used by the cancer research community. ‘Epithelial–mesenchymal transition’ became the term of use after the first TEMTIA meeting, which brought the field together in 2003.

Many observations originating more than a quarter century ago described EMT as being induced by a diverse array of contextual signals. For example, cultured amnion cells were ‘transformed’ from an epithelial phenotype into fibroblast-like cells in response to a leukocyte medium^17^. Endocardial cells underwent EMT in response to signals from adjacent cardiac muscle^18^. Hepatocyte growth factor (HGF) was found to induce transformation of epithelial cells into migratory fibroblasts^19^. Fibroblast growth factor 1 (FGF1) induced an ‘epithelial plasticity’ response in bladder carcinoma^10^, connecting EMT to cancer. TGFβ, overexpressed in cancers and required for cardiac EMT^20^, was found to be a potent inducer of EMT in cultured cells^11^. These observations provided the first indications that diverse extracellular signals, including soluble factors and components of the extracellular matrix (ECM), could act together to evoke EMT programmes in responding epithelial cells.

These early descriptive studies shed little light on the mechanisms operating within individual cells that enable the induction of EMT. Discoveries in the field of *Drosophila melanogaster* developmental genetics led to the identification of master regulators of EMT — for example, the transcription factors Snail and Twist, which act pleiotropically to orchestrate mesoderm formation during gastrulation^21^. The identification of related transcription factors in chordates revealed the high degree of conservation of these factors during metazoan evolution^22^ and thus highlighted the importance and relevance of studying various developmental animal model systems in order to understand EMT regulation.

Research aimed at identifying molecular regulators of EMT began on a large scale in the 1990s. For example, identification of the Snail-related transcription factor Slug (also known as Snai2) as an inducer of EMT during chick gastrulation and neural crest cell formation illustrated that specific transcription factors can act as key upstream regulators of EMT^23^. The finding that Slug expression can convert epithelial carcinoma cells into mesenchymal derivatives made a strong case for a connection between embryonic EMT and cancer progression^24^. This notion was reinforced by the observation that the Snail family of transcription factors are capable of inducing EMT and invasiveness (the capacity to leave the epithelial tissue and migrate into the underlying tissue) in normal epithelial cells, in part through transcriptional repression of the gene encoding E-cadherin^25–28^. Additional EMT-TFs, notably E47, Twist1, Zeb1 and Zeb2, were identified by means of their ability to evoke morphological and molecular changes associated with EMT^29–32^. It is important to note that these EMT-TFs usually cooperate with one another to orchestrate EMT. A large number of studies have also revealed that EMT-inducing signals can regulate the expression and activity of these EMT-TFs, doing so via both transcriptional and post-transcriptional mechanisms.

As our understanding of the inductive signals and transcriptional control of EMT evolved, it became apparent that the activation and execution of EMT does not require changes in DNA sequence and can be reversible. This made it clear, in turn, that EMT occurs as a result of complex epigenetic regulatory programmes, much like those operating at different stages of development. During development, some cell populations may undergo multiple rounds of EMT and MET, indicating substantial phenotypic plasticity. For example, during renal morphogenesis, the epithelial cells lining renal vesicles are derived from renal mesenchymal cells via MET, while these mesenchymal cells in turn are descendants of epithelial cells in the epiblast via EMT^33^. During somite formation, paraxial mesenchyme cells undergo MET to form epithelial somites, which then undergo EMT to give rise to the sclerotome^34^. Likewise, during the pathogenesis of cancers and fibrosis, EMT is activated to various degrees (from partial to fully) and is often reversible, revealing a plasticity that can yield cells residing in a spectrum of states, between a fully epithelial phenotype and a fully mesenchymal phenotype^3^ (considered as the end points of EMT). Thus, EMT does not result in a single mesenchymal state, but rather in a variety of intermediate states with various degrees of epithelial and mesenchymal features. This finding presents a major challenge to the EMT research community: how best to capture the diversity and plasticity of the EMT programmes operating in various biological contexts.

## EMT in development, cancer and fibrosis

A driving paradigm for the growth of this research field has been that EMT operates in normal tissues during development and wound healing, but is also a driver in the pathogenesis of cancer and fibrosis. The common starting point of diverse EMTs is the downregulation of certain features of the epithelial phenotype. Importantly, however, although it is now recognized that the EMT programmes do not operate as binary switches that shunt cells from fully epithelial to fully mesenchymal extremes, it remains unclear whether discrete phenotypic states are arrayed along the epithelial-to-mesenchymal (E-to-M) phenotypic spectrum or, alternatively, a continuum of such states exist that lack distinct, definable boundaries. The extent to which such intermediates represent stable states in specific biological contexts is also unclear. A continuum of EMT intermediate states might enable rapid interconversion between cells possessing various combinations of these traits, a process viewed as having high phenotypic plasticity. Moreover, it is possible that the phenotypic states between the fully epithelial and fully mesenchymal end points might not be arrayed along a linear spectrum, and that multiple alternative paths can operate to enable an epithelial cell to advance towards a mesenchymal state. Finally, the cells activating EMT programmes in adult tissues under pathological conditions commonly express combinations of epithelial and mesenchymal markers and rarely complete the entire EMT programme, suggesting that ‘partial EMTs’ represent the norm rather than the exception.

### Development

During animal development, cells of epithelial origin often migrate long distances from their original position to their final destination. The exit and detachment from the epithelial sheet and the following migration to distant locations depend on cells acquiring a mesenchymal state through EMT. Moreover, in many cases, the cells later undergo a permanent or temporary reversal to an epithelial state through MET (for example, endoderm cells) or switch to a different state (for example, neural crest cells). A great degree of morphological variability is associated with epithelial cells that participate in developmental EMTs, ranging from cells possessing fully formed epithelial cell–cell junctions (including tight junctions, adherens junctions and/or desmosomes) and an underlying basement membrane (to which they adhere through hemidesmosomes), such as the pluripotent epiblast cells of amniotes^35^, to the primitive epithelial cells giving rise to mesendoderm in *Xenopus laevis* and zebrafish, which exhibit only apical–basal polarity and incompletely assembled cell–cell junctions^36^.

In many cases, the quasi-mesenchymal state is not reached through a complete loss of cell–cell junctions, but instead by changes in the nature and dynamics of junction formation and dissolution, which may explain how cells with mesenchymal characteristics can exhibit collective cell migration^37^ — that is, the migration of cohorts of cells that seem to be held together by various types of cell–cell junctions. Such plastic, quasi-mesenchymal phenotypes are observed in cells that migrate collectively and are held together partially by cadherin-based cell–cell contacts, in endoderm and mesoderm cells of *D. melanogaster*, zebrafish, *X. laevis* and mouse^38–43^, and in neural crest cells of zebrafish, chick and *X. laevis*^44,45^. It is important to note that not every migratory process employed by epithelial cells involves EMT, as is the case in chicken epiblast morphogenesis before the formation of the primitive streak^46^.

### Cancer

During the multistep progression of carcinomas that are initially benign, epithelial cells acquire a few distinctly mesenchymal traits that confer to them the ability to invade adjacent tissues, locally, and then to disseminate to distant tissues. Much of this phenotypic progression towards increased invasiveness depends on the activation of EMT^3,47–51^. Carcinoma cells might be able to perform collective migration locally without activating EMT, possibly using collective migration mechanisms similar to those used during development. However, it is unclear whether primary carcinoma cells can complete the entire process of metastatic dissemination without activating, at least transiently, components of the EMT programme. The behaviour of carcinoma cells that transition to intermediate epithelial/mesenchymal states (E/M states) (that is, partial EMTs) echoes the behaviour of epithelial cells during normal development. Cancer cells proceed through a gradation of phenotypic states, each associated with combinations of epithelial and mesenchymal markers^3,51,52^.

The activation of alternative EMT programmes and the progression of individual cells to different states along the E-to-M spectrum can generate extensive phenotypic heterogeneity within tumours. Supporting this notion, multiple E/M cell subpopulations with distinct chromatin landscapes and gene expression signatures have been reported in skin and mammary primary tumours, and these subpopulations are often spatially localized within specific areas of a tumour^53^. Moreover, hybrid E/M states are enriched in circulating tumour cells (CTCs) that are released by primary breast and lung cancers and their metastases^54,55^, ostensibly reflecting the cellular heterogeneity seen within the originating primary tumours. Such phenotypic plasticity and heterogeneity may provide cancer cells with increased adaptability and resistance, enabling them to respond to a variety of external cues and physiological stresses^3,49,51,52,56,57^. Thus, because tumour cells encounter diverse microenvironments as they navigate the multiple steps of the metastatic cascade (Box 1) and migrate through and reach different tissues, various hybrid E/M phenotypes may provide a survival advantage in these distinct environments, such as blood and lymphatic vessels and primary and secondary tumour sites. The tissue of origin of the tumour cell, specific combinations of expressed EMT-TFs, and chromatin modifications may also determine the phenotypic heterogeneity of these various hybrid E/M states.

The diversity of EMT-associated cancer cell phenotypes is reflected in the discrepancies in experimental and histopathological observations of human tumours that have fueled a long-standing debate regarding the roles of EMT in cancer progression^4,5,58–60^. Such discrepancies can often be attributed to the use of different EMT markers and the analysis of particular EMT-TFs as markers of this programme; in addition, the EMT programmes operating in different tissues might differ from one another. For instance, Snai1 and Twist1 have both been shown to be important for metastasis in the PyMT-driven breast cancer model^61,62^, but dispensable for metastasis in a pancreatic cancer model^7^, which instead depends on Zeb1 (ref.^63^). Further confusion has come from the observation that carcinoma cells undergoing a partial EMT can reduce their epithelial phenotype through post-translational mechanisms^64^, making it challenging to interpret studies that rely solely on the perturbation of transcriptomes by EMT-TFs. These and other examples indicate that the versions of EMT programmes and the functions of the involved EMT-TFs are tissue context dependent. Moreover, as is often seen during the course of embryonic development, cancer-associated EMT is only activated partially and transiently, making end-stage markers of a fully mesenchymal state uninformative in cancer studies. Another complicating factor is that EMT programmes have been linked to additional traits that are not associated with canonical EMT regulation, such as stemness, cell survival rate, metabolic changes and, in the case of cancer cells, resistance to anticancer therapeutic drugs^65,66^.

Box 1 The metastasis cascade The metastatic process is thought to consist of the following sequential steps (see the figure). Initial escape from the primary site (invasion) requires that the epithelial tumour cells loosen their cell–cell junctions to become motile (step 1) and that they degrade the basement membrane and extracellular matrix (ECM); breakdown of these physical barriers allows cells to migrate and invade into nearby normal tissues (step 2). The next step of metastasis is termed ‘intravasation’, during which tumour cells invade across the endothelial lamina, penetrate into the vascular or lymphatic vessels and thereby enter the systemic circulation (step 3). Only a small number of the released neoplastic cells appear to be capable of surviving attack by the shear forces encountered in the circulation and by anoikis (a form of programmed cell death that occurs when cells detach from the surrounding ECM) provoked by the loss of anchorage to solid ECM. Eventually, some of the survivors may arrest in and extravasate through the capillary endothelium of distant organs into the parenchyma of these organs (extravasation) (step 4). In the new stromal environment that they encounter, an even smaller subset succeed in establishing themselves (step 5) and in proliferating from minute growths (micrometastases) into fully malignant secondary tumours that are clinically detectable and eventually life-threatening (secondary growth) (step 6). The activation of epithelial–mesenchymal transition (EMT) can provide tumour cells with the ability to migrate, invade, intravasate and extravasate. Once they reach distant organs, these mesenchymal cells revert to a more epithelial identity via mesenchymal–epithelial transition (MET) in order to regain proliferating ability, to form secondary growth in distant organs. Figure adapted from ref.^93^, CC BY 4.0 (https://creativecommons.org/licenses/by/4.0/).
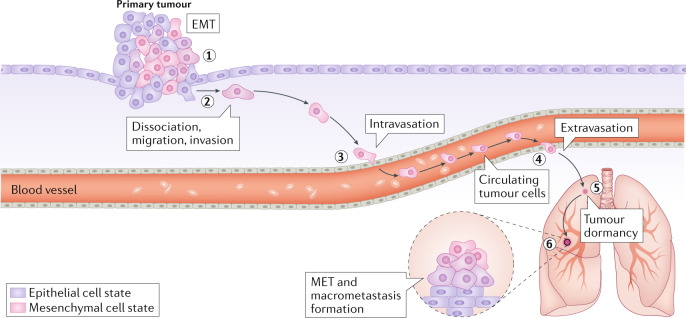

### Fibrosis

EMT has also been observed to occur and play a role in diverse types of fibrosis (including in the lung, liver and kidney), with EMT-TF expression shown to be a prerequisite for fibrosis development in mouse models^3^. As in cancer progression, the role of EMT in organ fibrosis has been the subject of active debate. A central issue in this debate is the origin of the myofibroblasts that accumulate in fibrotic tissues. These cells represent a specialized fibroblast population involved in collagen secretion and thus in the development and progression of interstitial fibrosis, which is the major cause of the disease in different tissues. Early lineage-tracing studies supported the hypothesis that myofibroblasts arise from EMT-driven conversion^67^, but subsequent lineage-tracing analyses have not provided compelling evidence of epithelial cells as precursors of fibrosis-associated myofibroblasts^68^. More recent studies have shown that renal epithelial cells undergo a partial EMT that is crucial for disease progression, but that they do not directly contribute to the formation of the myofibroblast population^68,69^. Instead, they lose their normal tubular function, and these damaged cells release paracrine signals to the renal interstitium, reshaping the microenvironment. The release of TGFβ converts existing fibroblasts into myofibroblasts, and the secretion of additional cytokines and chemokines probably recruits macrophages to the stroma. Hence, damaged renal epithelial cells promote both fibrogenesis and inflammation, which are hallmarks of renal fibrosis^69,70^. While the debate concerning the contribution of EMT to different types of fibrosis continues, the demonstrated requirement of EMT-TF expression strongly suggests that the activation of EMT is indeed required for the development of several types of fibrosis.

## Definitions of EMT and its associated terms

To facilitate the investigation of multifaceted EMT processes and discussion among diverse groups of researchers studying EMT, we propose the following definitions of EMT and its associated terms to stand as a reference. We encourage researchers to adhere to this recommended nomenclature.

### EMT

A multifaceted and often reversible change in cellular phenotypes during which epithelial cells lose their apical–basal polarity, modulate their cytoskeleton and exhibit reduced cell–cell adhesive properties. Cells may individually or collectively acquire mesenchymal features and increase motility and invasive ability. Typically, a switch in intermediate filament usage from cytokeratins to vimentin is observed after a complete EMT. Cortical actin filament in epithelial cells also undergoes marked rearrangement during EMT. While the characteristics of fully epithelial cells are relatively clearly defined, our current knowledge does not allow us to define the mesenchymal state with specific cellular characteristic or molecular markers that are universal end-products of all EMT programmes.

### MET

Reciprocal changes in cellular phenotype that reverse EMT-induced phenotypes, during which mesenchymal-like cells may acquire apical–basal polarity, reorganize their cytoskeleton, and exhibit increased cell–cell adhesion, resulting in an organized epithelium. MET occurs during embryonic development (for example, cardiac development, kidney morphogenesis and somite formation) and cancer.

### Endothelial–mesenchymal transition

As with epithelial cells, endothelial integrity depends on cell–cell junctions, apical–basal polarity and interactions with an underlying basement membrane. Endothelial–mesenchymal transition (EndoMT) more accurately indicates the phenomenon in such cell populations and resembles EMT in most aspects, except for the replacement of E-cadherin by VE-cadherin. EndoMT thereby enables endothelial cells to attenuate or deconstruct their functional integrity and apical–basal polarity, to acquire motile and invasive behaviour and to activate changes in gene expression that are driven by certain EMT-TFs. Similar to epithelial cells in EMT, endothelial cells that have activated EndoMT programmes exhibit a variety of intermediate or partial phenotypes, as discussed above for EMT. EndoMT was described initially during embryonic heart development^71^ and subsequently in the context of cardiac fibrosis^72^.

### Epithelial–mesenchymal plasticity

We favour and recommend use of the term ‘epithelial–mesenchymal plasticity’ (EMP) to describe the ability of cells to adopt mixed E/M features and to interconvert between intermediate E/M phenotypic states arrayed along the epithelial–mesenchymal spectrum that cannot be easily distinguished on the basis of our current understanding. This plasticity has been variably referred to as partial EMT, hybrid E/M status, a metastable EMT state, EMT continuum and EMT spectrum; in all cases, the cells express a mixture of epithelial features (such as cytokeratins) and mesenchymal features (such as cell migration) and markers. EMP indicates an ability to move readily between these various states, although the stability of the various states varies in different biological contexts. EMP is widely observed in development, wound healing and cancer. In addition to a mesenchymal type of migration, as observed during mesoderm formation, EMP can also participate in collective migration — for example, during tubulogenesis and wound healing. EMP also accounts for the reversibility of the EMT programme. Epithelial cells going through EMT give rise to cell populations that may enter reversibly into states with various proportions of epithelial and mesenchymal features. EMP is thought to provide cells with the fitness and flexibility to fulfil the diverse requirements during the course of either developmental or pathological processes.

### EMT-TFs

In many if not most settings, both in cell culture and in vivo, EMP involves some degree of transcriptional regulation. Several transcription factors belonging to the Snail, Twist and Zeb families have been found to control cell–cell adhesion, cell migration and ECM degradation, and to play evolutionarily conserved central roles in the execution of EMT in various biological settings and organisms (Table 1). Of note, all the developmental EMT processes described to date involve at least one member of these families of core EMT-TFs. Other transcription factors have been shown to impact EMT in certain contexts (Supplementary Table 1). However, these transcription factors are also involved in other cellular processes (for example, proliferation, apoptosis or stemness). In addition, many of the EMT-TFs are also expressed in non-epithelial cells, ranging from fibroblasts to haematopoietic precursors, and in cancer types involving non-epithelial derivatives (melanoma, glioblastoma and leukaemia), where they play important roles during tumour progression, often beyond classic EMT phases. Although we use the term EMT-TFs to describe all transcription factors associated with EMT, it is important to keep in mind that their expression alone is not sufficient to indicate that EMT is occurring.

**Table 1:** Core EMT transcription factors, with key studies reporting their discovery

Transcription factor	Type	Development	Cancer	Fibrosis
Snai1 (Snail)	Zinc finger	Boulay et al., 1987 (ref.^84^); Nieto et al., 1992 (ref.^85^)	Batlle et al., 2000 (ref.^25^); Cano et al., 2000 (ref.^26^)	Boutet et al., 2006 (ref.^86^)
Snai2 (Slug)	Zinc finger	Nieto et al., 1994 (ref.^23^)	Savagner et al., 1997 (ref.^24^)	—
Zeb1	Zinc finger	Funahashi et al., 1993 (ref.^87^)	Grooteclaes and Frisch, 2000 (ref.^88^)	Oba et al., 2010 (ref.^89^)
Zeb2 (SIP1)	Zinc finger	Verschueren et al., 1999 (ref.^90^)	Comijn et al., 2001 (ref.^31^)	Oba et al., 2010 (ref.^89^)
Twist1	bHLH	Thisse et al., 1988 (ref.^91^)	Yang et al., 2004 (ref.^30^)	Kida et al., 2007 (ref.^92^); Lovisa et al., 2015 (ref.^70^)

EMT, epithelial–mesenchymal transition.

## Recommendations on the criteria to define EMT

In the current EMT literature, both cellular and molecular descriptors have been used to define EMT in various biological systems. Below we provide several key recommendations on how to better use this information to cover the enormous complexity and plasticity of the EMT programme in diverse developmental and pathological settings.

### EMT status cannot be assessed on the basis of one or a small number of molecular markers

EMT constitutes changes of cell behaviour that involve the loss of certain epithelial characteristics and the gain of certain mesenchymal traits. The complex series of cellular changes occurring during EMT require the cooperation of a large number of molecular factors. On the basis of their involvement in the process, these factors can be divided into three groups: EMT-inducing signals, EMT-TFs and EMT markers that define and constitute various epithelial and mesenchymal cell characteristics. In the literature, diverse cellular and molecular descriptors have been used to define EMT in different biological systems, which has been a major source of confusion. For example, some studies define partial loss of E-cadherin as an indication of EMT, while others argue that the maintenance of certain levels of expression of epithelial markers such as cytokeratins is indicative of cells not having undergone EMT. Given the complex manifestations of the EMT programme, it has become clear that inferring the involvement of EMT in any process cannot rely solely on a few salient molecular markers, such as E-cadherin and vimentin^73^.

More importantly, the use of various EMT molecular markers to characterize the phenotypic state of individual tumour cells has revealed that such cells, as described earlier, can simultaneously express both epithelial and mesenchymal genes. The core EMT-TFs are often co-expressed in various combinations in order to orchestrate complex EMT programmes, and they involve various members of EMT-TF families, such as Snai (Snai1 and Snai2) and Zeb (Zeb1 and Zeb2), depending on the specific biological context^74^. Importantly, post-transcriptional regulation of EMT regulators at both the mRNA and protein levels is crucial in controlling EMT. Such regulation is often neglected in studies that use RNA expression exclusively to survey EMT molecular markers. A focus on defining EMT programmes exclusively on the basis of the expression of specific molecular markers such as these underrepresents the enormous complexity and plasticity of the EMT programmes in diverse developmental and pathological settings.

### The primary criteria for defining EMT status should be changes in cellular properties together with a set of molecular markers, rather than relying solely on molecular markers

One major feature that unites all the variant EMT programmes is the initial attenuation or deconstruction, to varying degrees and with diverse manifestations, of the epithelial phenotype. Epithelial cells harbour complexes that mediate cell–cell interactions, most notably adherens junctions, tight junctions and desmosomes (Fig. 1). Apical–basal polarity guides the proper organization of tight junctions, adherens junctions and desmosomes in epithelial cells. Polarity complexes, including the Par, Crumbs and Scribble complexes^75–77^, define the apical-lateral and basal-lateral domains of epithelial cells (Fig. 1). During the early phase of EMT, loss of apical–basal polarity is often the first event to be observed and can lead to the destabilization of adhesion complexes, such as the tight junctions and adherens junctions at the lateral membrane^78,79^, as well as to the activation of EMT-TFs^80^. The decrease or loss of epithelial adherens junctions and desmosomes occurs via transcriptional repression by the core EMT-TFs of the genes encoding junctional proteins. The cytoplasmic relocalization of adherens junction proteins, such as E-cadherin, via post-transcriptional regulation is also an early feature of EMT initiation in various EMT models^81^.

Another key function of the EMT programmes is to provide stationary epithelial cells with the ability to migrate by invading through extracellular matrices secreted by both epithelial and mesenchymal cells. Thus, during EMT, epithelial cells often need to breach the basement membrane in order to migrate away from their epithelium of origin^35,82^. Migration of cells that have undergone EMT does not necessarily require the cells to lose all epithelial features, and a switch of intermediate filaments from cytokeratin to vimentin can facilitate cell migration. Depending on the extent of cell–cell adhesion loss, epithelial cells can migrate as single cells, in a mesenchymal manner, or collectively, while remaining attached with one another via weakened but still operative cell–cell interactions. This indicates that the breakdown of these tightly regulated epithelial structures, the gain of motility and the ability to degrade ECM during EMT are inaccurately represented by the simple expression, or lack of expression, of selected markers. Furthermore, complex post-translational modifications of key proteins play critical roles in governing the complex cellular processes that occur during EMT. For these reasons, researchers should describe EMT and MET as functional changes in the biological properties of cells rather than focusing largely on changes in a few readily monitored molecular markers. It is through this perspective that our understanding of EMT could faithfully reflect the function of EMT during animal development and pathological events (Fig. 1). Therefore, whenever it is experimentally feasible, EMT should be assessed through the combination of cellular properties and multiple molecular markers.

### EMT-TFs and other molecular markers are valuable indicators of EMT, but they should be assessed in conjunction with changes in cellular characteristics to define EMT

The morphological and functional changes that can be observed in cells during EMT often result from changes in gene expression. Many, but not all, EMT-associated changes in gene expression result directly or indirectly from the actions of EMT-TFs, which play key roles in driving EMT. Indeed, most EMT programmes are associated with the activation of expression of one or several core EMT-TFs. Although core EMT-TFs often initiate EMT-associated changes in gene expression (Table 1), a large number of other EMT-TFs and numerous microRNAs and long non-coding RNAs (lncRNAs) have also been shown to contribute to or play critical roles in diverse EMTs (Supplementary Table 1). Decreased association between β-catenin or p120-catenin and E-cadherin, achieved by post-translational modifications, can also greatly weaken the adhesive functions of adherens junctions. Reduced expression of junctional and polarity proteins is often visible during EMT. Depending on the cell type and the extent to which cells advance through an EMT programme, cells undergoing an EMT may begin to express vimentin, to suppress cytokeratin, to shift expression of key integrins and so forth. These changes in gene expression are often seen as being indicative of EMT or as markers of EMT, although, considering the extensive variations in EMT, their overall value in the diagnosis of EMT needs to be considered with caution. Beyond this small set of commonalities, it is difficult to define other changes as contributing universally to all the diverse manifestations of EMT programmes that have been described in the rapidly expanding literature. Furthermore, our current knowledge does not allow us to know whether there is a linear succession of cell-biological changes as cells advance progressively through an EMT programme, or whether a diverse series of routes radiates in multiple directions from the starting point of attenuation or loss of epithelial junctions.

### Finding reliable EMT markers requires a combinatorial approach, as well as distinguishing between EMT-associated and non-EMT-associated functions

Analysing the state of a cell that is engaged in EMT often requires the use of markers that are specific to a specific biological context. To be able to assess in which position on the EMT spectrum a cell resides, it might be necessary to use a set of criteria that are standardized for the specific biological context. Obtaining quantitative EMT marker measurements should always be coupled with cellular and functional analyses of EMT status, as described above. Importantly, recent studies have linked EMT to various other cellular programmes and functions, including cancer cell stemness, resistance to apoptosis, genome instability, cancer drug resistance and metabolic adaptation. Many components of EMT regulatory pathways, including EMT-TFs, also affect other important cellular functions and phenotypes and are themselves regulated through diverse signals that may or may not involve canonical EMT. For example, EMT-TFs such as Snai1/2 and Twist1 also regulate cancer cell survival and cancer drug resistance^66,83^, and it is currently unknown whether cell survival and drug resistance are regulated independently of EMT. Likewise, many ECM remodeling proteins that are important for breaching the basement membrane can be regulated in both EMT-dependent and EMT-independent manners. Furthermore, although cells may switch to a different cell fate upon EMT, the EMT-associated cellular changes from epithelial to more mesenchymal phenotypes are independent of cell differentiation or cell dedifferentiation. Therefore, it is important to note that the cell-biological definition of EMT strictly refers to cellular features describing the E-to-M phenotypes, while we should keep these associated phenotypes in mind when examining the role of EMT in various biological settings.

## Implications for future EMT research

Fifty years and over 38,000 publications after Betty Hay’s pioneering observations, the concept of EMT has now been widely applied in biomedical research. It provides a unifying framework for developmental and cancer studies, which is evidenced by the exponentially growing number of EMT-related publications. Such a framework holds the promise of far-reaching breakthroughs in cancer diagnosis and treatment, for cancer biologists, and of bridging the gap in understanding normal and pathological epithelial organization and morphogenesis, for developmental and cell biologists. To realize this promise, it is desirable that the EMT community reach a consensus on the definition of EMT-related terms and on the conceptual framework for approaching EMT as a biological process with quantifiable molecular descriptors and cellular readouts. All the authors of this Consensus Statement article have agreed to adhere to the recommendations on nomenclature presented here in their future research publications and recommend that other researchers in the EMT and the larger biological research communities also follow these guidelines. Only by minimizing semantic misinterpretation and data miscommunication can we begin to appreciate the diversity of individual EMTs and uncover conserved themes in EMT regulation between development and disease.

Studies using cell lines, developmental systems and cancer models have revealed a diversity of EMT-induced phenotypes and have highlighted remarkable complexity in the execution and regulation of EMT. Looking forward, to decipher the complexity and plasticity of the EMT programme, we propose that EMT research, while remaining anchored in traditional developmental, cell and cancer biology, should be explored within a broader conceptual context. The EMT field has in recent years attracted the interest of a diverse group of researchers with expertise in systems biology, biophysics, stem cell biology, pathology and mathematical modelling. This remarkable strength of interest will enable cross-disciplinary collaborations and push this field of research forward. We expect that future EMT studies will apply multidisciplinary approaches in order to gain increased mechanistic understanding of EMT. One open question in the cancer EMT field is the extent to which the stabilization of specific hybrid E/M states, or the dynamic switch between E/M states in response to distinct cues from the microenvironment, favours the metastatic process. With many important aspects of EMT remaining unexplored, advancing EMT research will require technological innovations to enable the study of both developmental and cancer-associated EMT at the single-cell level. These innovations will include single-cell live imaging, lineage tracing, gene expression analyses and studies of genetic and epigenetic modifications. Finally, a combination of mathematical modelling with carefully constructed experimental analyses will be important to gaining a mechanistic understanding of EMT plasticity.

Another major challenge is the translation of the current knowledge of EMT heterogeneity and plasticity into clinical practice. While we are far from understanding the functional implications of EMT heterogeneity, several clinical trials have already incorporated the notion of EMT plasticity^3^, thereby opening the way for novel therapies that exploit EMT heterogeneity. Single-cell sequencing of normal tissues, primary tumours, circulating tumour cells and metastases, combined with cellular analyses and functional validations, will capture the diversity and plasticity of EMT and have the potential to reveal the molecular alterations underlying tumour progression and the diverse responses to therapy. An increased understanding of the EMT mechanisms associated with these behaviours offers the potential for targeted therapy to prevent cancer metastasis. For example, while the inhibition of EMT-associated changes might reduce cancer cell dissemination in early-stage carcinoma, preventing MET in disseminated tumour cells might inhibit metastatic outgrowth in distant organs. Experimental and clinical studies have shown that the development of resistance to various therapies, including chemotherapies and immunotherapies, is tightly associated with EMT phenotypes^83^. These studies suggest that targeting EMT, or the cells capable of executing it, holds promise in overcoming therapy resistance, which is a major challenge in cancer treatment.

## Supplementary information

Supplementary Information

## Glossary

Neural crest: A multipotent cell population formed at the interface between the neuroepithelium and the epidermis. Neural crest cells give rise to multiple cell types, including the neurons, glial cells, pigment cells, fibroblasts, smooth muscle cells, odontoblasts and adipocytes.
Müllerian duct: A coelomic epithelium-derived tubular structure present in early reproductive tract development. Müllerian duct gives rise to the oviduct, uterus, cervix and part of the vagina in the female embryo. It degenerates in the male embryo through the action of anti-Müllerian hormone.
Renal vesicles: Epithelialized vesicles derived from the metanephric mesenchyme during kidney development. Each renal vesicle gives rise to one nephron and, together with vascular-derived glomerulus and ureteric bud-derived collecting duct, forms the basic unit of renal filtration.
Epiblast: A population of pluripotent cells during early mammalian, reptilian and avian development. The epiblast undergoes epithelialization, initiates gastrulation morphogenesis and gives rise to the three principal germ layers (ectoderm, mesoderm and endoderm).
Somite: An epithelial vesicle formed through periodic budding from the mesenchymal pre-somitic mesoderm. The somite is located between the neural tube and the intermediate mesoderm and gives rise to the sclerotome (axial bones) and dermomyotome (dermis and skeletal muscles).
Sclerotome: A part of the epithelial somite that undergoes EMT first and gives rise to the vertebrae and ribs.
Tight junctions: Intercellular barriers formed between epithelial cells. Such junctions regulate transepithelial particle transport and prevent the free diffusion of cell membrane proteins between the apical and basolateral domains.
Adherens junctions: A type of cell–cell junction in which the plasma membranes of apposing cells adhere to each other through cadherin-mediated homophilic interactions. Adherens junctions associate with condensed actin filaments intracellularly.
Desmosomes: A type of cell–cell junction that provides strong adhesion between cells and resists mechanical stress. Desmosomes interact with intermediate filaments intracellularly.
Hemidesmosomes: A type of cell–matrix attachment structure mediated by integrin–matrix interactions extracellularly and intermediate filaments intracellularly.
PyMT: A breast cancer metastasis model in which the polyomavirus middle T-antigen is overexpressed in mammary epithelial cells as a driver of breast tumour development.
Interstitial fibrosis: Excessive deposition of extracellular matrix by fibroblasts in interstitial space, leading to tissue hardening and scarring.
Renal interstitium: The renal parenchymal space that is outside the tubular, glomerular and vascular structures.
Fibrogenesis: The phenomenon of excessive production of extracellular matrix by fibroblasts.
Cancer cell stemness: The presence of subpopulations of cancer cells that exhibit stem cell-like behaviour.
Dedifferentiation: The conversion of cells from a more differentiated state to a less differentiated one. Dedifferentiation is often associated with the acquisition of progenitor or stem cell-like properties.
