# Author Correction: Guidelines and definitions for research on epithelial–mesenchymal transition

**DOI:** 10.1038/s41580-021-00428-9

**Published:** 2021-10-15

**Authors:** Jing Yang, Parker Antin, Geert Berx, Cédric Blanpain, Thomas Brabletz, Marianne Bronner, Kyra Campbell, Amparo Cano, Jordi Casanova, Gerhard Christofori, Shoukat Dedhar, Rik Derynck, Heide L. Ford, Jonas Fuxe, Antonio García de Herreros, Gregory J. Goodall, Anna-Katerina Hadjantonakis, Ruby Y. J. Huang, Chaya Kalcheim, Raghu Kalluri, Yibin Kang, Yeesim Khew-Goodall, Herbert Levine, Jinsong Liu, Gregory D. Longmore, Sendurai A. Mani, Joan Massagué, Roberto Mayor, David McClay, Keith E. Mostov, Donald F. Newgreen, M. Angela Nieto, Alain Puisieux, Raymond Runyan, Pierre Savagner, Ben Stanger, Marc P. Stemmler, Yoshiko Takahashi, Masatoshi Takeichi, Eric Theveneau, Jean Paul Thiery, Erik W. Thompson, Robert A. Weinberg, Elizabeth D. Williams, Jianhua Xing, Binhua P. Zhou, Guojun Sheng, On behalf of the EMT International Association (TEMTIA)

**Affiliations:** 1 Departments of Pharmacology and Pediatrics, Moores Cancer Center, University of California, San Diego, La Jolla, CA USA; 2 Department of Cellular and Molecular Medicine, University of Arizona, Tucson, AZ USA; 3 Molecular and Cellular Oncology Lab, Department of Biomedical Molecular Biology, Ghent University, Cancer Research Institute Ghent (CRIG), VIB Center for Inflammation Research, Ghent, Belgium; 4 Laboratory of Stem Cells and Cancer, Université Libre de Bruxelles, Bruxelles, Belgium; 5 Department of Experimental Medicine 1, Nikolaus-Fiebiger-Center for Molecular Medicine, Friedrich-Alexander-University Erlangen-Nürnberg, Erlangen, Germany; 6 Division of Biology and Biological Engineering, California Institute of Technology, Pasadena, CA USA; 7 Department of Biomedical Science and Bateson Centre, University of Sheffield, Sheffield, UK; 8 Departamento de Bioquímica, Universidad Autónoma de Madrid (UAM), Instituto de Investigaciones Biomédicas ‘Alberto Sols’ (CSIC-UAM), IdiPAZ & Centro de Investigación Biomédica en Red de Cáncer (CIBERONC), Madrid, Spain; 9 Institute for Research in Biomedicine (IRB Barcelona), Barcelona Institute of Science and Technology/Institut de Biologia Molecular de Barcelona (IBMB-CSIC), Barcelona, Spain; 10 Department of Biomedicine, University of Basel, Basel, Switzerland; 11 Department of Biochemistry and Molecular Biology, University of British Columbia and British Columbia Cancer Research Centre, Vancouver, BC Canada; 12 Departments of Cell and Tissue Biology, and Anatomy, University of California at San Francisco, San Francisco, CA USA; 13 Department of Pharmacology, University of Colorado Anschutz Medical Campus, Aurora, CO USA; 14 Department of Laboratory Medicine (LABMED), Division of Pathology, Karolinska University Hospital and Department of Microbiology, Tumor and Cell Biology (MTC), Karolinska Institutet, Stockholm, Sweden; 15 Programa de Recerca en Càncer, Institut Hospital del Mar d’Investigacions Mèdiques (IMIM) and Departament de Ciències Experimentals i de la Salut, Universitat Pompeu Fabra, Barcelona, Spain; 16 Centre for Cancer Biology, An alliance of SA Pathology and University of South Australia, Adelaide, SA Australia; 17 Developmental Biology Program, Sloan Kettering Institute, Memorial Sloan Kettering Cancer Center, New York, NY USA; 18 School of Medicine, College of Medicine, National Taiwan University, Taipei, Taiwan; 19 Department of Medical Neurobiology, Institute for medical Research Israel-Canada and the Safra Center for Neurosciences, Hebrew University of Jerusalem, Hadassah Medical School, Jerusalem, Israel; 20 Department of Cancer Biology, Metastasis Research Center, MD Anderson Cancer Center, Houston, TX USA; 21 Department of Molecular Biology, Princeton University, Princeton, NJ USA; 22 Centre for Cancer Biology, an Alliance of SA Pathology and the University of South Australia, Adelaide, SA Australia; 23 Department of Physics, Northeastern University, Boston, MA USA; 24 Department of Anatomic Pathology, The Division of Pathology and Laboratory Medicine, University of Texas MD Anderson Cancer Center, Houston, TX USA; 25 Department of Medicine (Oncology) and Department of Cell Biology and Physiology, ICCE Institute, Washington University, St. Louis, MO USA; 26 Department of Translational Molecular Pathology, University of Texas MD Anderson Cancer Center, Houston, TX USA; 27 Cancer Biology and Genetics Program, Sloan Kettering Institute, Memorial Sloan Kettering Cancer Center, New York, NY USA; 28 Department of Cell and Developmental Biology, University College London, London, UK; 29 Department of Biology, Duke University, Durham, NC USA; 30 Departments of Anatomy and Biochemistry/Biophysics, University of California, San Francisco, School of Medicine, San Francisco, CA USA; 31 Murdoch Children’s Research Institute, Royal Children’s Hospital, Parkville, VIC Australia; 32 Instituto de Neurociencias (CSIC-UMH) Avda Ramon y Cajal s/n, Sant Joan d´Alacant, Spain; 33 Université Claude Bernard Lyon 1, INSERM 1052, CNRS 5286, Centre Léon Bérard, Cancer Research Center of Lyon, Lyon, France; 34 Department of Cellular and Molecular Medicine, University of Arizona, Tucson, AZ USA; 35 INSERM UMR 1186, Integrative Tumor Immunology and Genetic Oncology, Gustave Roussy, University Paris-Saclay, Villejuif, France; 36 Department of Medicine, Perelman School of Medicine, University of Pennsylvania, Philadelphia, PA USA; 37 Department of Zoology, Graduate School of Science, Kyoto University, Kyoto, Japan; 38 RIKEN Center for Biosystems Dynamics Research, Kobe, Japan; 39 Centre de Biologie du Développement (CBD), Centre de Biologie Intégrative (CBI), Université de Toulouse, CNRS, UPS, Toulouse, France; 40 Guangzhou Regenerative Medicine and Health, Guangdong Laboratory, Guangzhou, China; 41 School of Biomedical Sciences and Institute of Health and Biomedical Innovation, Queensland University of Technology, Translational Research Institute, Woolloongabba, QLD Australia; 42 Whitehead Institute for Biomedical Research, Department of Biology, MIT Ludwig Center for Molecular Oncology, Massachusetts Institute of Technology, Cambridge, MA USA; 43 Australian Prostate Cancer Research Centre-Queensland (APCRC-Q) and Queensland Bladder Cancer Initiative (QBCI), School of Biomedical Sciences and Institute of Health and Biomedical Innovation, Queensland University of Technology, Woolloongabba, QLD Australia; 44 Department of Computational and Systems Biology and UPMC-Hillman Cancer Center, University of Pittsburgh, Pittsburgh, PA USA; 45 Department of Molecular and Cellular Biochemistry and UK Markey Cancer Center, University of Kentucky College of Medicine, Lexington, KY USA; 46 International Research Center for Medical Sciences (IRCMS), Kumamoto University, Kumamoto, Japan; 47 Present Address: Institut Curie, PSL Research University, Paris, France

**Keywords:** Epithelial-mesenchymal transition

Correction to: *Nature Reviews Molecular Cell Biology* 10.1038/s41580-020-0237-9, published online 16 April 2020.

In the supplementary information, the reference to Contreras et al. 2016 was removed (as it relates to a gene different from E2-2/TCF4), and ‘(TCF7L2)’ was deleted from the table. Misspellings in the author name Ruby Y. J. Huang and in the PyMT glossary definition were also corrected. These changes have been made to the HTML and PDF versions of the article.

