# Supplementary material for: Guidelines and definitions for research on epithelial–mesenchymal transition
Source: Nat Rev Mol Cell Biol. 2020 Apr 16;21(6):341–52. doi: 10.1038/s41580-020-0237-9 (PMC7250738; doi:10.1038/s41580-020-0237-9)
Supplement: Supplementary file 1 — Supplementary Information [file 41580_2020_237_MOESM1_ESM.pdf]

nature portfolio

https://doi.org/10.1038/s41580-020-0237-9

Supplementary information

Guidelines and definitions for research on epithelial–mesenchymal transition

In the format provided by the authors and unedited

Supplementary Table:
Additional transcription factors associated with EMT and key studies reporting their discovery in the context of development, cancer and fibrosis.

Transcription factor*	Type	Development	Cancer	Fibrosis
**Alx1**	Homeobox	Saunders & McClay, 2014	Yuan et al., 2013	-
**AP1 (Jun/Fos Atf)**	Ap1	Reichmann et al., 1992	Bakiri et al. 2015	Alcorn et al., 2009
**Brachyury (T)**	T-box	Beddington et al., 1992	Fernando et al., 2010	Sun et al., 2014
**CBF-A (NF-YA)**	CCAT box binding	-	Venkov et al., 2007	Venkov et al., 2007
**E2-2 (TCF4)**	bHLH	Sobrado et al., 2009	Sobrado et al., 2009	-
**E47 (TCF3)**	bHLH	Perez-Moreno et al., 2001	Perez-Moreno et al., 2001	Slattery et al., 2006
**Erg**	ETS	Saunders & McClay, 2014	Petrovics et al., 2005	-
**Ets1**	ETS	Fafeur et al., 1997	Nakayama et al., 1996	Nakamura et al., 2004.
**FoxC2 (FoxB)**	Forkhead	Saunders & McClay, 2014	Mani et al., 2007	Sipos & Galamb, 2012
**GATA4/6 (Srp)**	GATA	Campbell et al, 2011	-	-
**Goosecoid (GSC)**	Homeobox	Blum et al., 1992	Hartwell et al., 2006	-
**HoxB7**	Homeobox	-	Wu et al, 2006	-
**Id1**	HLH	Jen et al., 1996	Tobin et al, 2011	Li et al., 2007
**KLF2**	Sp/KLF	Chiplunkar et al., 2013	-	-
**Klf4**	Klf	Liu et al., 2012	Lin et al., 2012	Tiwari et al., 2013
**KLF6 (Z9f)**	Sp/KLF	-	-	Holian et al., 2008
**KLF8**	Sp/KLF	-	Wang et al, 2007	-
**NF-κb**	NF-κB/Rel	DeLaughter et al., 2016	Huber et al, 2004	Sunami et al., 2012
**Prrx1**	Paired related homeobox	Ocana et al. 2012	Ocana et al., 2012; Takano et al., 2016	Gong et al., 2017
**Runx2**	Runt-related	Tavares et al. 2017	Pratap et al., 2005	Mummler et al., 2018

**Six1**	Homeobox	Grifone et al., 2005	Micalizzi et al., 2009; McCoy et al., 2009	Smith et al., 2008
**Sox4**	Homeobox	Zhang et al, 2012	Liu et al., 2006	Xiao et al., 2015
**Sox9**	Homeobox	Akiyama et al., 2004	Acevedo et al., 2007	Hanley et al., 2008
**Tbx3**	T-box	Singh et al., 2012	Rodriguez et al. 2008	Wensing & Campos, 2014
**Tead (Yap/Taz)**	Tead	Diepenbruck et al., 2014	Overholtzer et al., 2006	Seo et al 2016
**Tgif1**	Homeobox	Saunders & McClay, 2014	Xiang et al., 2015	-
**Twist2 (Dermo1)**	bHLH	-	Ansieau et al., 2008	Grunz-Borgmann et al., 2017

*Transcription factors with a minimum of 10 citations in the EMT literature. "–" indicates lack of a pertinent reference.

References:

Acevedo, V.D., Gangula, R. D., Freeman, K. W., Li, R. L., Zhang, Y. Y., Wang, F., Ayala, G. E., Peterson, L. E., Ittmann, M. & Spencer, D. M. Inducible FGFR-1 activation leads to irreversible prostate adenocarcinoma and an epithelial-to-mesenchymal transition. Cancer Cell 12, 559-571 (2007).

Akiyama, H., Chaboissier, M. C., Behringer, R. R., Rowitch, D. H., Schedl, A., Epstein, J. A. & de Crombrugghe, B. Essential role of Sox9 in the pathway that controls formation of cardiac valves and septa. Proc Natl Acad Sci U S A 101, 6502-6507 (2004).

Alcorn, J.F., van der Velden, J., Brown, A. L., McElhinney, B., Irvin, C. G. & Janssen-Heininger, Y. M. W. c-Jun N-Terminal Kinase 1 Is Required for the Development of Pulmonary Fibrosis. American Journal of Respiratory Cell and Molecular Biology 40, 422-432 (2009).

Ansieau, S., Bastid, J., Doreau, A., Morel, A. P., Bouchet, B. P., Thomas, C., Fauvet, F., Puisieux, I., Doglioni, C., Piccinin, S., Maestro, R., Voeltzel, T., Selmi, A., Valsesia-Wittmann, S., Caron de Fromentel, C. & Puisieux, A. Induction of EMT by twist proteins as a collateral effect of tumor-promoting inactivation of premature senescence. Cancer Cell 14, 79-89 (2008).

Bakiri, L., Macho-Maschler, S., Custic, I., Niemiec, J., Guio-Carrion, A., Hasenfuss, S. C., Eger, A., Muller, M., Beug, H. & Wagner, E. F. Fra-1/AP-1 induces EMT in mammary epithelial cells by modulating Zeb1/2 and TGF beta expression. Cell Death Differ 22, 336-350 (2015).

Beddington, R.S.P., Rashbass, P.R. & Wilson, V. Brachyury--a gene affecting mouse gastrulation and early organogenesis. Development.-.Supplement., 157-165 (1992).

Blum, M., Gaunt, S. J., Cho, K. W., Steinbeisser, H., Blumberg, B., Bittner, D., & De Robertis, E. M. Gastrulation in the mouse: the role of the homeobox gene goosecoid. Cell 69, 1097-1106 (1992).

Campbell, K., Whissell, G., Franch-Marro, X., Batlle, E. & Casanova, J. Specific GATA factors act as conserved inducers of an endodermal-EMT. *Dev Cell* **21**, 1051-1061, doi:10.1016/j.devcel.2011.10.005 (2011)

Chiplunkar, A.R., Lung, T. K., Alhashem, Y., Koppenhaver, B. A., Salloum, F. N., Kukreja, R. C., Haar, J. L. & Lloyd, J. A. Kruppel-like factor 2 is required for normal mouse cardiac development. Plos One 8, e54891 (2013).

DeLaughter, D.M., Clark, C. R., Christodoulou, D. C., Seidman, C. E., Baldwin, H. S., Seidman, J. G. & Barnett, J. V. Transcriptional Profiling of Cultured, Embryonic Epicardial Cells Identifies Novel Genes and Signaling Pathways Regulated by TGFbetaR3 In Vitro. Plos One 11, e0159710 (2016).

Diepenbruck, M., Waldmeier, L., Ivanek, R., Berninger, P., Arnold, P., van Nimwegen, E.& Christofori, G. Tead2 expression levels control the subcellular distribution of Yap and Taz, zyxin expression and epithelial-mesenchymal transition. Journal of Cell Science 127, 1523-1536 (2014).

Fafeur, V., Tulasne, D., Queva, C., Vercamer, C., Dimster, V., Mattot, V., Stehelin, D., Desbiens, X. & Vandenbunder, B. The ETS1 transcription factor is expressed during epithelial-mesenchymal transitions in the chick embryo and is activated in scatter factor-stimulated MDCK epithelial cells. Cell Growth & Differentiation 8, 655-665 (1997).

Fernando, R.I., Litzinger, M., Trono, P., Hamilton, D. H., Schlom, J. & Palena, C. The T-box transcription factor Brachyury promotes epithelial-mesenchymal transition in human tumor cells. J Clin Invest 120, 533-544 (2010).

Gong, J., Han, J., He, J. Y., Liu, J. M., Han, P., Wang, Y. W., Li, M. K., Li, D. X., Ding, X. M., Du, Z. P., Liao, J. Z. & Tian, D. Paired related homeobox protein 1 regulates PDGF-induced chemotaxis of hepatic stellate cells in liver fibrosis. Laboratory Investigation 97, 1020-1032 (2017).

Grifone, R., Demignon, J., Houbron, C., Souil, E., Niro, C.,. Seller, M. J., Hamard, G. & Maire, P. Six1 and Six4 homeoproteins are required for Pax3 and Mrf expression during myogenesis in the mouse embryo. Development 132, 2235-2249 (2005).

Grunz-Borgmann, E.A., Nichols, L.A., Wang, X.H. & Parrish, A.R. Twist2 Is Upregulated in Early Stages of Repair Following Acute Kidney Injury. Int J Mol Sci 18 (2017).

Hanley, K.P., Oakley, F., Sugden, S., Wilson, D. I., Mann, D. A. & Hanley, N. A. Ectopic SOX9 mediates extracellular matrix deposition characteristic of organ fibrosis. Journal of Biological Chemistry 283, 14063-14071 (2008).

Hartwell, K.A., Muir, B., Reinhardt, F., Carpenter, A. E., Sgroi, D. C. & Weinberg, R. A. The Spemann organizer gene, Goosecoid, promotes tumor metastasis. Proc Natl Acad Sci U S A 103, 18969-18974 (2006).

Holian, J., Qi, W., Kelly, D. J., Zhang, Y., nMreich, E., Pollock, C. A. & Chen, X. M. Role of Kruppel-like factor 6 in transforming growth factor-beta1-induced epithelial-mesenchymal transition of proximal tubule cells. Am J Physiol Renal Physiol 295, F1388-1396 (2008).

Huber, M.A., Azoitei, N., Baumann, B., Grunert, S., Sommer, A., Pehamberger, H., Kraut, N., Beug, H. & Wirth, T. NF-kappa B is essential for epithelial-mesenchymal transition and metastasis in a model of breast cancer progression. Journal of Clinical Investigation 114, 569-581 (2004).

Jen, Y., Manova, K. & Benezra, R. Expression patterns of Id1, Id2, and Id3 are highly related but distinct from that of Id4 during mouse embryogenesis. Developmental Dynamics 207, 235-252 (1996).

Li, Y., Yang, J., Luo, J.H., Dedhar, S. & Liu, Y. Tubular epithelial cell dedifferentiation is driven by the helix-loop-helix transcriptional inhibitor Id1. J Am Soc Nephrol 18, 449-460 (2007).

Lin, Z.S., Chu, H.C., Yen, Y.C., Lewis, B.C. & Chen, Y.W. Kruppel-like factor 4, a tumor suppressor in hepatocellular carcinoma cells reverts epithelial mesenchymal transition by suppressing slug expression. Plos One 7, e43593 (2012).

Liu, P.B., Ramachandran, S., Ali Seyed, M., Scharer, C. D., Laycock, N., Dalton, W. B., Williams, H., Karanam, S., Datta, M. W., Jaye, D. L. & Moreno, C. S. Sex-determining region Y box 4 is a transforming oncogene in human prostate cancer cells. Cancer Research 66, 4011-4019 (2006).

Liu, Y.N., Abou-Kheir, W., Yin, J. J., Fang, L., Hynes, P., Casey, O., Hu, D., Wan, Y., Seng, V., Sheppard-Tillman, H., Martin, P. & Kelly, K. Critical and reciprocal regulation of KLF4 and SLUG in transforming growth factor beta-initiated prostate cancer epithelial-mesenchymal transition. Mol Cell Biol 32, 941-953 (2012).

Mani, S.A., Yang, J., Brooks, M., Schwaninger, G., Zhou, A., Miura, N., Kutok, J. L., Hartwell, K., Richardson, A. L. & Weinberg, R. A. Mesenchyme Forkhead 1 (FOXC2) plays a key role in metastasis and is associated with aggressive basal-like breast cancers. Proceedings of the National Academy of Sciences of the United States of America 104, 10069-10074 (2007).

McCoy, E.L., Iwanaga, R., Jedlicka, P., Abbey, N. S., Chodosh, L. A., Heichman, K. A., Welm, A. L. & Ford, H. L.Six1 expands the mouse mammary epithelial stem/progenitor cell pool and induces mammary tumors that undergo epithelial-mesenchymal transition. J Clin Invest 119, 2663-2677 (2009).

Micalizzi, D.S., Christensen, K. L., Jedlicka, P., Coletta, R. D., Baron, A. E., Harrell, J. C., Horwitz, K. B., Billheimer, D., Heichman, K. A., Welm, A. L., Schiemann, W. P.& Ford, H. L. The Six1 homeoprotein induces human mammary carcinoma cells to undergo epithelial-mesenchymal transition and metastasis in mice through increasing TGF-beta signaling. Journal of Clinical Investigation 119, 2678-2690 (2009).

Mummler, C., Burgy, O., Hermann, S., Mutze, K., Gunther, A., & Konigshoff, M. Cell-specific expression of runt-related transcription factor 2 contributes to pulmonary fibrosis. Faseb Journal 32, 703-716 (2018).

Nakamura, Y., Esnault, S., Maeda, T., Kelly, E. A. B., Malter, J. S. & Jarjour, N. N. Ets-1 regulates TNF-alpha-induced matrix metalloproteinase-9 and tenascin expression in primary bronchial fibroblasts. Journal of Immunology 172, 1945-1952 (2004).

Nakayama, T., Ito, M., Ohtsuru, A., Naito, S., Nakashima, M., Fagin, J. A., Yamashita, S. & Sekine, I. Expression of the Ets-1 proto-oncogene in human gastric carcinoma - Correlation with tumor invasion. American Journal of Pathology 149, 1931-1939 (1996).

Ocana, O.H., Corcoles, R., Fabra, A., Moreno-Bueno, G., Acloque, H., Vega, S., Barrallo-Gimeno, A., Cano, A. & Nieto, M. A. Metastatic Colonization Requires the Repression of the Epithelial-Mesenchymal Transition Inducer Prrx1. Cancer Cell 22, 709-724 (2012).

Overholtzer, M., Zhang, J., Smolen, G. A., Muir, B., Li, W., Sgroi, D. C., Deng, C. X., Brugge, J. S. & Haber, D. A. Transforming properties of YAP, a candidate oncogene on the chromosome 11q22 amplicon. Proceedings of the National Academy of Sciences of the United States of America 103, 12405-12410 (2006).

Perez-Moreno, M.A., Locascio, A., Rodrigo, I., Dhondt, G., Portillo, F., Nieto, M. A. & Cano, A. A new role for E12/E47 in the repression of E-cadherin expression and epithelial-mesenchymal transitions. Journal of Biological Chemistry 276, 27424-27431 (2001).

Petrovics, G., Liu, A. J., Shaheduzzaman, S., Furasato, B., Sun, C., Chen, Y. M., Nau, M., Ravindranath, L., Chen, Y. D., Dobi, A., Srikantan, V., Sesterhenn, I. A., McLeod, D. G., Vahey, M., Moul, J. W. & Srivastava, S. Frequent overexpression of ETS-related gene-1 (ERG1) in prostate cancer transcriptome. Oncogene 24, 3847-3852 (2005).

Pratap, J., Javed, A., Languino, L. R., van Wijnen, A. J., Stein, J. L., Stein, G. S.

& Lian, J. B. The Runx2 Osteogenic Transcription Factor Regulates Matrix Metalloproteinase 9 in Bone Metastatic Cancer Cells and Controls Cell Invasion. Molecular and Cellular Biology 25, 8581-8591 (2005).

Reichmann, E., Schwarz, H., Deiner, E. M., Leitner, I., Eilers, M., Berger, J., Busslinger, M. & Beug, H. Activation of an inducible c-FosER fusion protein causes loss of epithelial polarity and triggers epithelial-fibroblastoid cell conversion. Cell 71, 1103-1116 (1992).

Rodriguez, M., Aladowicz, E., Lanfrancone, L. & Goding, C.R. Tbx3 represses E-cadherin expression and enhances melanoma invasiveness. Cancer Research 68, 7872-7881 (2008).

Saunders, L.R. & McClay, D.R. Sub-circuits of a gene regulatory network control a developmental epithelial-mesenchymal transition. Development 141, 1503-1513 (2014).

Seo, E., Kim, W. Y., Hur, J., Kim, H., Nam, S. A., Choi, A., Kim, Y. M., Park, S. H., Chung, C., Kim, J., Min, S., Myung, S. J., Lim, D. S. & Kim, Y. K. The Hippo-Salvador signaling pathway regulates renal tubulointerstitial fibrosis. Scientific Reports 6 (2016).

Singh, R. et al. Tbx2 and Tbx3 induce atrioventricular myocardial development and endocardial cushion formation. Cell Mol Life Sci 69, 1377-1389 (2012).

Sipos, F. & Galamb, O. Epithelial-to-mesenchymal and mesenchymal-to-epithelial transitions in the colon. World J Gastroentero 18, 601-608 (2012).

Slattery, C., McMorrow, T. & Ryan, M.P. Overexpression of E2A proteins induces epithelial-mesenchymal transition in human renal proximal tubular epithelial cells suggesting a potential role in renal fibrosis. Febs Letters 580, 4021-4030 (2006).

Smith, J.C., Boone, B.E., Opalenik, S.R., Williams, S.M. & Russell, S.B. Gene profiling of keloid fibroblasts shows altered expression in multiple fibrosis-associated pathways. Journal of Investigative Dermatology 128, 1298-1310 (2008).

Sobrado, V.R., Moreno-Bueno, G., Cubillo, E., Holt, L. J., Nieto, M. A., Portillo, F. & Cano, A. The class I bHLH factors E2-2A and E2-2B regulate EMT. Journal of Cell Science 122, 1014-1024 (2009).

Sun, S., Sun, W., Xia, L., Liu, L., Du, R., He, L., Li, R., Wang, H & Huang, C. The T-box transcription factor Brachyury promotes renal interstitial fibrosis by repressing E-cadherin expression. Cell Commun Signal 12, 76 (2014).

Sunami, Y., Leithauser, F., Gul, S., Fiedler, K., Guldiken, N., Espenlaub, S., Holzmann, K. H., Hipp, N., Sindrilaru, A., Luedde, T., Baumann, B., Wissel, S., Kreppel, F., Schneider, M., Scharffetter-Kochanek, K., Kochanek, S., Strnad, P. & Wirth, T. Hepatic activation of IKK/NFkappaB signaling induces liver fibrosis via macrophage-mediated chronic inflammation. Hepatology 56, 1117-1128 (2012).

Tavares, A.L.P., Brown, J.A., Ulrich, E.C., Dvorak, K. & Runyan, R.B. Runx2-I is an Early Regulator of Epithelial-Mesenchymal Cell Transition in the Chick Embryo. Dev Dyn (2017).

Tiwari, N., Meyer-Schaller, N., Arnold, P., Antoniadis, H., Pachkov, M., van Nimwegen, E. & Christofori, G. Klf4 Is a Transcriptional Regulator of Genes Critical for EMT, Including Jnk1 (Mapk8). Plos One 8 (2013).

Tobin, N.P., Sims, A.H., Lundgren, K.L., Lehn, S. & Landberg, G. Cyclin D1, Id1 and EMT in breast cancer. Bmc Cancer 11, 417 (2011).

Venkov, C.D., Link, A. J., Jennings, J. L., Plieth, D., Inoue, T., Nagai, K., Xu, C., Dimitrova, Y. N., Rauscher, F. J. & Neilson, E. G. A proximal activator of transcription in epithelial-mesenchymal transition. Journal of Clinical Investigation 117, 482-491 (2007).

Wang, X., Zheng, M., Liu, G., Xia, W., McKeown-Longo, P. J., Hung, M. C. & Zhao, J. Kruppel-like factor 8 induces epithelial to mesenchymal transition and epithelial cell invasion. Cancer Res 67, 7184-7193 (2007).

Wensing, L.A. & Campos, A.H. TBX3, a downstream target of TGF-beta 1, inhibits mesangial cell apoptosis. Experimental Cell Research 328, 340-350 (2014).

Wu, X., Chen, H., Parker, B., Rubin, E., Zhu, T., Lee, J. S., Argani, P. & Sukumar, S. HOXB7, a homeodomain protein, is overexpressed in breast cancer and confers epithelial-mesenchymal transition. Cancer Res 66, 9527-9534 (2006).

Xiang, G., Yi, Y., Weiwei, H. & Weiming, W. TGIF1 promoted the growth and migration of cancer cells in nonsmall cell lung cancer. Tumour Biol 36, 9303-9310 (2015).

Xiao, L., Zhou, X., Liu, F. Y., Hu, C., Zhu, X. J., Luo, Y., Wang, M., Xu, X. X., Yang, S. K., Kanwar, Y. S. & Sun, L. MicroRNA-129-5p modulates epithelial-to-mesenchymal transition by targeting SIP1 and SOX4 during peritoneal dialysis. Laboratory Investigation 95, 817-832 (2015).

Yuan, H., Kajiyama, H., Ito, S., Yoshikawa, N., Hyodo, T., Asano, E., Hasegawa, H., Maeda, M., Shibata, K., Hamaguchi, M., Kikkawa, F. & Senga, T. ALX1 induces snail expression to promote epithelial-to-mesenchymal transition and invasion of ovarian cancer cells. Cancer Res 73, 1581-1590 (2013).

Zhang, J., Liang, Q., Lei, Y., Yao, M., Li, L., Gao, X., Feng, J., Zhang, Y., Gao, H., Liu, D. X., Lu, J. & Huang, B. SOX4 induces epithelial-mesenchymal transition and contributes to breast cancer progression. Cancer Res 72, 4597-4608 (2012).
